# Application of Machine Learning in Amorphous Alloys

**DOI:** 10.3390/ma18081771

**Published:** 2025-04-13

**Authors:** Like Zhang, Huangyou Zhang, Boyan Ji, Leqing Liu, Xianlan Liu, Ding Chen

**Affiliations:** 1 College of Intelligent Manufacturing and Mechanical Engineering, Hunan Institute of Technology, Hengyang 421002, China; 2 College of Mechanical and Vehicle Engineering, Hunan University, Changsha 410082, China

**Keywords:** machine learning, amorphous alloys, glass-forming ability, properties

## Abstract

In the past few decades, traditional methods for developing amorphous alloys, such as empirical trial-and-error approaches and density functional theory (DFT)-based calculations, have enabled researchers to explore numerous amorphous alloy systems and investigate their properties. However, these methods are increasingly unable to meet the demands of modern research due to their long development cycles and low efficiency. In contrast, machine learning (ML) has gained widespread adoption in the design, analysis, and property prediction of amorphous alloys due to its advantages of low experimental cost, powerful performance, and short development cycles. This review focuses on four key applications of ML in amorphous alloys: (1) prediction of amorphous alloy phases, (2) prediction of amorphous composite phases, (3) prediction of glass-forming ability (GFA), and (4) prediction of material properties. Finally, we outline future directions for ML in materials science, including the development of more sophisticated models, integration with high-throughput experimentation, and the creation of standardized data-sharing platforms. These insights provide potential research directions and frameworks for subsequent studies in this field.

## 1. Introduction

Amorphous alloys, also known as metallic glasses, are a class of novel materials developed since the 1960s [1,2]. The term “amorphous alloy” arises from the fact that during ultra-rapid cooling, atoms do not have sufficient time to arrange themselves into an ordered crystalline structure, resulting in a solid alloy with long-range disordered atomic arrangement. Due to the absence of defects such as dislocations, stacking faults, and grain boundaries, amorphous alloys exhibit exceptional properties in certain physical aspects, including high hardness, high strength, corrosion resistance, and wear resistance [3]. However, traditional methods have struggled to establish clear structure–property relationships, hindering a deeper understanding of the intrinsic nature of amorphous materials and impeding the design and development of novel high-performance amorphous alloys.

**Figure 1:**
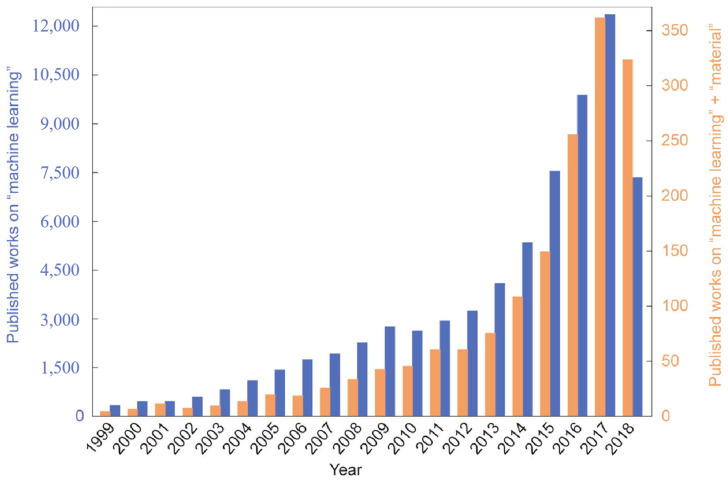
Number of published works on “machine learning” and “machine learning” + “material” (from January 1999 to September 2018) [7]. Copyright © 2019, Elsevier Ltd.

The concept of ML was first introduced by Samuel in 1959 [4]. As a crucial branch of artificial intelligence, ML enables the imitation of human learning processes through the utilization of extensive known data and algorithms, progressively improving performance to achieve accurate predictions on new data [5,6]. Figure 1 presents a statistical analysis of literature combining ML with materials science from 1999 to 2018, demonstrating that the integration of ML with materials research has become an inevitable trend over time [7]. With more than half a century of research, a substantial amount of experimental data on amorphous alloys has been accumulated, laying the foundation for the application of data-centric ML in this field and providing reliable Big Data support. In the context of Big Data, ML has been extensively applied in the field of amorphous alloys, significantly accelerating the design process of these materials. Currently, the application of ML in amorphous alloys primarily manifests in four aspects: (1) prediction of amorphous alloy phases, (2) prediction of amorphous composite phases, (3) prediction of GFA, and (4) prediction of amorphous alloy properties.

## 2. ML for Phase Prediction in Amorphous Alloys

To predict the phases of amorphous alloys, it is essential to collect data on alloy compositions, including amorphous alloys, high-entropy alloys (HEAs), and intermetallic compounds. In studying the influence of mixing entropy on the GFA of amorphous alloys, a class of HEAs with high mixing entropy was proposed in 2004 [8]. Since their introduction, HEAs with high mixing entropy have garnered significant attention from materials researchers. Yeh et al. [9] discovered the presence of solid solution phases in multi-component HEAs and further proposed that this phenomenon is related to the high mixing entropy of atoms in the alloy. To identify HEA compositions with superior mechanical properties from a vast pool of possibilities, the combination of multiple elements has become a focal point of research. Early HEAs exhibited low strength at room temperature, and their compositions [8,9] are typically referred to as single-phase solid solution alloys formed by random combinations of at least four principal elements in equimolar or near-equimolar ratios. Even when secondary phases were added, the goal was to enhance the mechanical properties of the solid solution alloys. Expanding the compositional space of HEAs traditionally required extensive trial-and-error methods, which were resource-intensive in terms of both time and materials. To rapidly identify amorphous alloys, solid solution alloys, and HEAs containing intermetallic compounds, ML, with its artificial intelligence (AI) capabilities, has emerged as an effective approach for exploring alloy phase selection. The advent of contemporary AI methods has the potential to fundamentally transform and enhance the role of computing in scientific and engineering fields. The integration of Big Data and AI has been termed the “Fourth Paradigm of Science” and the “Fourth Industrial Revolution”. As a branch of AI, ML has advanced rapidly in recent years. At its core, ML relies on statistical algorithms whose generalization performance can be improved through continuous training [10]. Generalization performance refers to the predictive capability of a learning model when tested on data governed by the same principles but with different unknown variables. ML is a computer algorithm that automatically improves based on general experience [11], aiming to optimize task performance through prior experience [12]. We are now in an era of breakthroughs in AI, which demands higher standards for the application of ML. Indeed, ML techniques and methods have already been successfully applied in numerous fields [13,14,15,16,17].

For the phase selection problem in HEAs, traditional experimental approaches rely heavily on trial-and-error methods, requiring substantial raw materials and phase testing. To reduce costs associated with raw materials and testing, ML can predict outcomes using collected data without the need for extensive additional experiments or simulations. In training ML models for HEA phase selection, the first step is to gather reported alloy data. The quality and quantity of data significantly influence the accuracy of predictions and the reliability of the model. For instance, Liu et al. [18] collected 3227 data points, including 1850 glassy materials and 1372 non-glassy materials, achieving a phase prediction accuracy of 83% using ML. Islam et al. [19] used 118 data points to predict phase selection among amorphous alloys, solid solution alloys, and intermetallic compounds, achieving a test set accuracy of 83%. These results indicate that both excessive and insufficient data can reduce prediction accuracy. Zhou et al. [20] collected 601 data points and predicted phases for amorphous, solid solution, and intermetallic compounds, achieving accuracies of 95.6%, 97.8%, and 92.2%, respectively. Huang et al. [21] gathered 401 data points, including 174 solid solution alloys, 54 intermetallic compounds, and 173 mixed solid solution and intermetallic compounds, with an average prediction accuracy of 94.3%. Zhang et al. [22] obtained 407 data points using a weighted removal method, reporting prediction accuracies of 88.77–94.63% for amorphous phases, 95.67–97.87% for solid solution phases, and 96.03–98.63% for mixed solid solution and intermetallic phases. Wu et al. [23] selected 321 data points, using 241 for training and 80 for testing, achieving a test set accuracy of 98.3%. These studies suggest that prediction accuracy is highest when the dataset size ranges between 300 and 600 [20,21,22,23,24,25].

Based on empirical and semi-empirical rules, many researchers have employed numerous feature parameters to study alloy phase selection. However, identifying the optimal combination of parameters for high prediction accuracy remains a topic of active discussion. Parameter combinations offer opportunities to fine-tune target properties [26], thereby establishing relationships between parameters and alloy phases and performance. Zhou et al. [20] selected 13 feature parameters, including atomic size difference (*δ*), average mixing enthalpy (Δ*H_m_*), mixing entropy (*S_id_*), and electronegativity difference (Δ*χ*), to predict the phase composition of amorphous alloys, solid solution alloys, and intermetallic compounds. The combination of Δ*H_m_* + *δ* improved accuracy to 75–93%, while *S_id_*+ Δ*H_m_* + *δ* + Δ*χ* achieved 84–95% accuracy. When all 13 parameters were used, prediction accuracy reached 92–98% [20]. Liu et al. [18] selected 11 feature parameters to predict phase selection between amorphous and crystalline alloys, achieving a lower accuracy of 83%. When studying the importance of these 11 parameters for GFA, they found that removing certain parameters, particularly *S_id_*, *δ*, Δ*H_m_*, and Δ*χ*, reduced prediction accuracy by over 7% [18], highlighting the critical role of these parameters. Islam et al. [19] used five feature parameters (valence electron concentration (*VEC*), *S_id_*, *δ*, Δ*H_m_*, and Δ*χ*)) to predict phases in amorphous alloys, solid solutions, and intermetallic compounds. Qu et al. [27] selected four thermodynamic parameters (*VEC*, *δ*, Δ*H_m_*, and Δ*χ*) to predict HEAs’ phase composition, achieving an accuracy of 95.75%. However, using too many feature parameters can reduce prediction accuracy during ML model training. The lack of unified physical significance in selected parameters often leads to random combinations or an excessive number of features, complicating the selection process.

In the selection of ML algorithms for phase prediction, researchers commonly employ artificial neural networks (ANNs), support vector machines (SVMs), naive Bayes algorithms, and K-nearest neighbors (KNN) algorithms. Zhou et al. [20] utilized ANNs, SVMs, and convolutional neural networks (CNNs) in their study. When using the ANN algorithm, the prediction accuracy was 98.9% for amorphous alloy phases, 95.6% for intermetallic compound phases, and 97.8% for solid solution phases. With the CNN algorithm, the prediction accuracy was 96.7% for amorphous alloy phases, 95.6% for intermetallic compound phases, and 98.9% for solid solution phases. When employing the SVM algorithm, the prediction accuracy was 97.8% for amorphous alloy phases, 94.4% for intermetallic compound phases, and 98.9% for solid solution phases [20]. These results demonstrate that all three ML algorithms achieved high prediction accuracy. In a separate study, Huang et al. [21] applied three ML algorithms—KNN, ANNs, and SVMs—to predict phase selection in alloys. By comparing the prediction accuracies of these algorithms, they found that the KNN algorithm achieved an accuracy of 68.6%, the SVM algorithm achieved 64.3%, and the ANN algorithm achieved 74.3%.

These findings indicate that ANNs generally provide higher prediction accuracy compared to SVMs. Syl et al. [28] compared four algorithms and concluded that neural networks outperformed the other three models, achieving a prediction accuracy of 85%. Similarly, Zhang et al. [22] compared SVMs, decision trees, and multilayer perceptrons, finding that SVMs achieved the highest prediction accuracy of 97.43%. Given the significant variability in prediction accuracy among different algorithms, some researchers have opted to use a single algorithm for predicting alloy phase selection. For instance, Zhang et al. [24] used only a genetic algorithm to predict phase selection in solid solutions, non-solid solutions, face-centered cubic (FCC), body-centered cubic (BCC), and dual-phase HEAs. By selecting the most suitable ML model and optimizing the combination of feature parameters, they achieved prediction accuracies of 88.7% for solid solution and non-solid solution HEAs and 91.3% for FCC, BCC, and dual-phase HEAs. Wu et al. [23] employed a single ANN algorithm to predict phase selection in eutectic HEAs, achieving a final test set accuracy of 98.3%. Similarly, Zhang et al. [29] used only a naive Bayes algorithm, achieving an impressive prediction accuracy of 99.6%. These results demonstrate that even when using a single ML algorithm, high prediction accuracy can be attained, provided the model and parameters are appropriately optimized.

## 3. ML for the Study of Multi-Principal Element Amorphous Alloy Composites

In the 21st century, the rapid development of fields such as aerospace, marine engineering, large-scale aircraft, high-speed rail equipment, and biomedicine has driven the need for novel high-performance and multifunctional amorphous alloy composites. The development of new alloys begins with the design of alloy components. The limitation of single-principal-element alloy design lies in the fact that, within a given compositional design space, the approach of adding elements to enhance properties restricts the exploration of new alloy compositions [30]. In 2004, the concept of multi-principal-element alloys broke through the limitations of traditional single-principal-element alloy design [9,31,32]. According to the definition of multi-principal-element alloys, early research focused on alloys based on configurational entropy and single-phase solid solutions [33]. Currently, multi-principal-element alloys encompass all alloy systems within the high-entropy alloy (HEA) domain, meeting the research demands for concentrated alloys or alloy systems with extensive compositional ranges and complex microstructures in the central regions of multi-principal-element phase diagrams [33].

Multi-principal-element amorphous alloys exhibit significant advantages over traditional metallic materials in terms of hardness, strength, toughness, thermal stability, electromagnetic properties, corrosion resistance, and radiation resistance, demonstrating high academic value and industrial potential [34,35,36,37,38,39]. When intermetallic compounds are introduced into eutectic alloy compositions with multiple principal elements, the intermetallic compounds can nucleate first during the cooling of the alloy melt [40]. To suppress the rapid growth of single-crystal phases and control the grain size of crystalline phases, other competing crystalline phases must nucleate and grow within the alloy melt. When the grains of competing crystalline phases mutually inhibit rapid growth, small-grained multi-phase amorphous alloy composites can be prepared. When the content of the second phase reaches 35–40%, the plasticity and work-hardening capacity of the amorphous alloy composites can be improved [41]. Therefore, the compositional design of novel multi-principal-element amorphous alloy composites requires not only eutectic units but also the inclusion of intermetallic compounds. The approach of “mixing intermetallic compound components with eutectic units” for designing multi-phase amorphous alloy composites can avoid deposition in single-crystal phases, and the atomic percentages of alloy components must avoid the traditional dominance of a single principal element [42]. The composition of multi-principal-element amorphous alloy composites can satisfy the requirements of competitive deposition among alloy components while retaining some amorphous structure. Alloy compositions designed by mixing intermetallic compound components and binary eutectic units in varying proportions represent a class of multi-phase, multi-principal-element amorphous alloy composites.

ML is a flexible and highly nonlinear modeling approach based on available data. Methods relying on physical principles often struggle to handle the complex relationships between material composition, phase structure, and properties. As a result, ML has become an essential tool for predicting material properties, screening compositions, and optimizing design [18,43,44,45,46,47,48,49,50,51]. In the prediction of phase selection for multi-principal-element alloys, Islam et al. [19] achieved a prediction accuracy of 83% using a neural network model. Mitra et al. [52] used the K-nearest neighbors (KNN) algorithm to predict single-phase versus multi-phase structures, achieving a multi-phase prediction accuracy of 98%. Prediction accuracy varies with the choice of algorithm; for instance, Hou et al. [53] proposed integrating empirical knowledge models into the prediction process, achieving an accuracy of over 83.3%. Among 13 selected feature parameters, different parameter combinations yield varying prediction accuracies for HEA phase selection. In an ANN model, Zhou et al. [20] reported prediction accuracies of 98.9% for amorphous phases, 95.6% for intermetallic phases, and 97.8% for solid solution phases, highlighting the importance of feature parameters based on potential energy distribution functions. Thus, ML demonstrates high accuracy in predicting phase selection for both multi-principal-element alloys and HEAs. However, when predicting optimal compositions for target properties, Li et al. [54] proposed a data-driven approach to accelerate the design of magnetic HEAs with combined saturation magnetization and hardness. Using ML, they developed a multi-objective optimization algorithm to identify optimal alloy compositions. In their study, the support vector regression (SVR) model achieved the lowest root mean square error (RMSE) of 13.9% for magnetization, while the gradient boosting decision tree model achieved the lowest RMSE of 57.6% for hardness prediction [54].

Feature parameter selection is a critical issue in alloy phase selection and property prediction. Too few parameters reduce prediction accuracy, while too many complicate the prediction process. Li et al. [55] proposed a feature selection method that effectively reduces the number of parameters without sacrificing accuracy. Using five models to predict magnetic properties and maximum critical size, the gradient boosting decision tree model achieved an *R*^2^ value of 0.93 for magnetic properties and 0.68 for maximum critical size. These results demonstrate the strong generalization performance of ML in alloy phase selection and property prediction.

ML is an effective method for predicting the composition, structure, and properties of multi-principal-element alloys. Typical regression algorithms include Gaussian process regression [56], artificial neural networks (ANNs) [57], and support vector machines (SVMs) [58]. In phase selection prediction, neural network models exhibit high accuracy, with atomic size playing a crucial role in the phase selection of multi-principal-element alloys [20,21,23,24,44,59,60,61,62,63]. For predicting amorphous, intermetallic, and solid solution phases, three parameters (atomic radius, electronegativity, and average mixing enthalpy) yield higher prediction accuracy (*Pc*) than four parameters (including mixing entropy) [64].

ML can not only predict the composition and GFA of multi-principal-element amorphous alloys [11,18,44] but also identify key parameters influencing the critical casting diameter of amorphous alloys [11], thereby enabling the prediction and design of multi-principal-element amorphous alloys [59].

## 4. ML for Predicting GFA

Although amorphous alloys exhibit excellent mechanical and chemical properties, their GFA remains a significant challenge hindering their development. If an alloy cools at a rate exceeding the critical cooling rate (the slowest cooling rate, Rc), it will not crystallize. Thus, the critical cooling rate is the most reliable indicator for measuring GFA. However, determining the critical cooling rate experimentally is often difficult. Instead, the critical diameter is commonly used to evaluate GFA, as it is easier to measure experimentally. Under identical cooling conditions, a larger critical diameter indicates stronger GFA. The relationship between cooling rate, critical diameter, and GFA can be summarized as follows: slower cooling rates result in larger critical diameters, thereby enhancing GFA [65]. Traditional physical methods for studying GFA rely on various material-specific and physics-based formulas, followed by experimental measurements of the maximum critical size, leading to an expensive and time-consuming trial-and-error process. While DFT or first-principles calculations can be used to predict phase selection or other properties, these methods fail when considering the wide range of critical sizes required for GFA evaluation. In contrast, ML offers a powerful solution to these complex problems.

In recent years, numerous researchers have made significant progress in predicting the GFA of amorphous alloys using ML. Common models for predicting GFA or critical diameter include random forests, artificial neural networks (ANNs), and extreme gradient boosting (XGBoost). Among these, random forest models have demonstrated robust performance in predicting critical diameters. For example, Xiong et al. [66] developed a Gaussian support vector machine (SVM) model using a dataset of 442 critical diameters, achieving an *R*^2^ value of 0.57, which, while significantly better than the *R*^2^ = 0.38, still falls short of practical application standards. Deng et al. [67] used the same dataset with a random forest model, improving the *R*^2^ value to 0.64, a 13% increase over the previous result. Xiong et al. [44] predicted GFA for 6471 alloys composed of 52 different elements, finding that the random forest model achieved the highest *R* value of 0.85 compared to symbolic regression. Ward et al. [49] constructed random forest, decision tree, and ensemble models using 607 amorphous alloy datasets, with the random forest model achieving 89% accuracy in predicting GFA for ternary and higher-order alloys, demonstrating excellent predictive performance. Liu et al. [68] collected a dataset of 6816 alloys, including 1027 amorphous alloys, and achieved 89% accuracy with a random forest model. Peng et al. [69] used 810 data points to build a random forest model, achieving an *R*^2^ value of 0.682, indicating strong generalization performance.

Li et al. [70] collected 5706 alloy compositions involving 51 elements and built classifier models, including decision trees, random forests, SVMs, logistic regression, and AdaBoost. The random forest model achieved over 88% accuracy, further confirming its reliability in predicting GFA. Extreme gradient boosting (XGBoost) models have also shown excellent generalization performance. For instance, Liu et al. [71] used 660 amorphous alloy datasets to compare four models (random forest, KNN, gradient boosting decision trees, and XGBoost) for predicting GFA based on critical casting diameter. The XGBoost model achieved the highest correlation coefficient (0.755) and the lowest root mean square error (*RMSE*) of 3.277 [71]. Xiong et al. [72] built an XGBoost model using 695 data points, achieving a correlation coefficient of 0.8012 for predicting GFA in alloys with Dmax ≥ 5 mm. Zhang et al. [73] developed five prediction models using 952 alloy datasets with critical diameters, with the XGBoost model achieving an R^2^ value of 0.8, outperforming the other four models.

Analyzing the relationship between composition and properties in amorphous alloys has long been challenging. To address this, Tripathi et al. [74] used a genetic algorithm on 410 datasets, achieving 95.26% accuracy in predicting GFA, demonstrating the effectiveness of genetic algorithms in this context. Mastropietro et al. [75] compared multiple linear regression and tree boosting models using 480 alloy datasets, finding that integrating these models achieved an R value of 0.84 for predicting GFA in iron-based amorphous alloys, indicating strong generalization performance [75]. Lu et al. [76] selected 663 samples involving 42 elements and found that an ANN model outperformed a CNN model in predicting critical diameters, achieving 77.623% accuracy. Reddy et al. [77] used 962 datasets to build ANN and KNN models for predicting critical diameters in 273 amorphous alloys, with the ANN model achieving an *R* value of 0.757. Liu et al. [78] collected 820 experimental datasets and used a gradient boosting decision tree model to predict GFA, achieving an R^2^ value of 0.652. Long et al. [79] built six models (decision tree, SVM regression, random forest, gradient boosting decision tree, KNN, and XGBoost) using 698 datasets involving 46 elements, with the decision tree model achieving an *R*^2^ value of 0.763 [79]. These results demonstrate that sufficient training data can enable accurate GFA prediction even for complex multi-element systems.

When predicting critical diameters for amorphous alloys with different elemental compositions, varying results are obtained. Sun et al. [46] used 91 datasets to build an SVM model for analyzing GFA in binary amorphous alloys, successfully predicting new alloys with excellent GFA. Xu et al. [80] developed six models (KNN, gradient boosting decision tree, SVM regression, random forest, ensemble learning, and XGBoost) using 840 alloy datasets to predict critical diameters in ternary amorphous alloys. The SVM regression model exhibited the best generalization performance, with maximum *R*^2^ values of 0.859 (test set) and 0.934 (training set) and minimum *RMSE* values of 0.56 and 0.4, respectively [80]. Hu et al. [81] collected 5316 compositions from 251 ternary alloy systems involving 52 elements and built four models (SVM, random forest, ANN, and ensemble learning). The ensemble learning model achieved an accuracy of 0.775, demonstrating strong generalization performance and effectively predicting GFA in ternary amorphous alloys. Xie et al. [82] used 7795 datasets to build four deep learning models based on recurrent neural networks (RNNs), achieving a test set accuracy of 0.843 and successfully predicting GFA in CuZrAl and CuCeGa ternary systems.

To address data imbalance issues, researchers have proposed strategies for data balancing. Yao et al. [83] collected 5725 datasets involving 1977 amorphous alloys with 20 elements and applied data balancing to an ANN model, improving prediction accuracy by 31% and enabling better GFA prediction for ternary amorphous alloys. Liu et al. [84] used 411 alloy datasets to build stacked and HiTp models, with the HiTp model demonstrating low cross-entropy loss and strong generalization performance for predicting GFA in ternary amorphous alloys. Samavatian et al. [85] developed a correlation-based neural network using 7950 alloy compositions to predict critical diameters in Zr-Co-Al-Ni quaternary amorphous alloys, achieving an *R* value of 0.95541 and identifying high-GFA quaternary systems. Tan et al. [86] collected 1016 critical diameter datasets involving 52 elements and built an ANN model to predict critical diameters for 439 unknown quaternary amorphous alloys in the Zr-Co-Al-X (X = W, Si, Ni) system.

## 5. ML for Predicting Other Properties of Amorphous Alloys

Liu et al. [87] built four ML models to predict the hardness of amorphous alloys, with the gradient boosting decision tree model achieving an *R*^2^ value exceeding 0.97, significantly outperforming the other models. Due to their disordered structure, amorphous alloys exhibit low magnetic properties, known as soft magnetism. Soft magnetic amorphous alloys have significant potential in electronic and electrical engineering applications, attracting considerable research attention.

**Figure 2:**
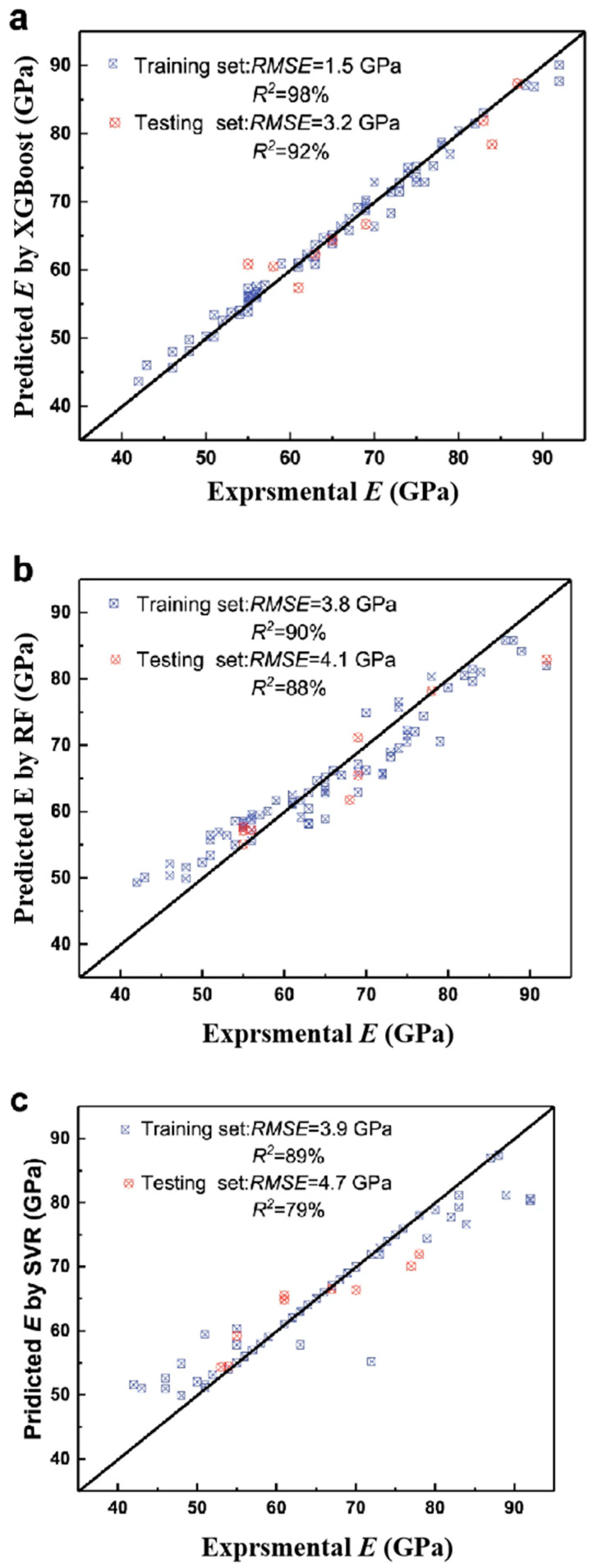
The relationship between the experimental Young’s modulus and the predicted values of the optimal model: (**a**) XGBoost; (**b**) RF; (**c**) SVR [90]. Copyright © 2020, Springer Nature.

Liu et al. [88] used Pearson correlation coefficients and recursive feature elimination to select seven parameters and built four models to predict the magnetic properties of amorphous alloys. The decision tree model achieved an *R*^2^ value of 0.9 and a mean absolute percentage error of 13.31%, demonstrating strong predictive performance. Tang et al. [89] developed models for predicting soft magnetic properties using linear regression, SVM regression, decision tree regression, ANN, and random forest regression. The ANN model achieved the highest *R*^2^ value, exceeding 0.9, and demonstrated excellent fitting capability for the soft magnetic properties of newly designed alloys [89]. Yang et al. [90] used gradient boosting decision tree, random forest, and SVM regression models to predict Young’s modulus. As shown in Figure 2, the gradient boosting decision tree model achieved the lowest *RMSE* (1.5 GPa) and highest *R*^2^ (98%) for the training set, and the lowest *RMSE* (3.2 GPa) and highest R^2^ (92%) for the test set. Zhu et al. [91] used ANN, random forest, KNN, and gradient boosting models to predict the yield strength and tensile strength of composite materials. The random forest model performed best, with *R*^2^ values of 0.92 for yield strength and 0.89 for tensile strength. These results highlight the significant advantages of ML in predicting the mechanical properties of amorphous alloys.

## 6. Comparison and Analysis

### 6.1. Phase Prediction and Multi-Principal Element Amorphous Alloy Composites

This paper compares the performance of machine learning models using different features and data volumes for prediction in Table 1, based on existing machine learning methods for phase prediction of multi-principal element amorphous alloy composites.

From the above comparison, it can be concluded that:

(1) If the amount of data is too large or too small, the prediction accuracy will decrease. When the number of data points is between 300 and 600, the prediction accuracy is higher.
(2) The lack of unified physical significance in selected parameters often leads to random combinations or an excessive number of features, complicating the selection process. Among them, *S_id_*, *δ*, Δ*H_m_*, and Δ*χ* are the four features that are more important.
(3) Various machine learning algorithms have different advantages and disadvantages, and it is necessary to choose the most suitable machine learning model and the optimal combination of feature parameters.

### 6.2. Predicting GFA and Predicting Other Properties

This paper compares and analyzes the existing predictions of amorphous alloys’ glass-forming ability and performance using machine learning in Table 2.

In summary, selecting appropriate machine learning algorithms to evaluate the glass-forming ability (GFA) and properties of amorphous alloys requires a holistic consideration of problem type, data characteristics, model performance, and interpretability requirements. Table 3 shows the advantages and limitations of several common machine learning algorithms.

(1) Problem Type Clarification. Classification tasks (e.g., predicting amorphous phase formation likelihood): Logistic regression, SVM, random forest, and XGBoost are recommended. Regression tasks (e.g., predicting quantitative GFA metrics such as critical cooling rate or glass-forming parameters): Linear regression, SVR, random forest regression, and XGBoost regression are suitable.
(2) Data Scale and Quality Assessment. Small sample sizes (<1000 samples): Prioritize models with low sample requirements (e.g., SVM, random forest). High-dimensional features: Apply feature selection (random forest-based importance, LASSO) or dimensionality reduction (e.g., PCA). Missing values/noise: Use robust algorithms (e.g., random forest) or implement data imputation/cleaning.
(3) Feature Engineering and Selection. Key features: Elemental composition, atomic size mismatch, thermodynamic parameters (e.g., mixing enthalpy, entropy), and kinetic parameters (e.g., undercooled liquid region width). Preprocessing: Standardization/normalization (critical for SVM/neural networks), generation of interaction/polynomial features.
(4) Model Selection and Optimization. Baseline models: Start with linear models (logistic/linear regression) to establish performance benchmarks. Nonlinear relationships: Random forest or XGBoost, which also enable feature importance analysis. High-dimensional data: SVM (requires hyperparameter tuning for kernel functions and regularization) with standardized inputs. Neural networks: Consider only for large datasets, with careful architecture design and regularization to prevent overfitting. Ensemble methods (e.g., Stacking): Improve performance at the cost of computational resources.
(5) Model Evaluation and Validation. Cross-validation: Use k-fold cross-validation (especially for small datasets) to mitigate overfitting. Metrics: Classification: Accuracy, F1-score (to address class imbalance), ROC–AUC. Regression: MSE, R^2^ score. Interpretability: Leverage SHAP values or LIME to explain complex models (e.g., XGBoost, neural networks).

Hu et al. [92] also reached similar conclusions through research, such as that the SVM model is most suitable for small datasets, and XGBoost is often used in machine learning competitions. At the same time, other researchers have also conducted research on amorphous alloy predictions. For example, Madanchi et al. [93] studied machine learning interatomic potential (MLIP), which has a lower cost than DFT and brings DFT-level accuracy to atomic simulations while improving accuracy and sampling efficiency. All of these provide support for the integration of machine learning into the research of amorphous alloys.

## 7. Summary and Outlook

In summary, the integration of ML into the study of amorphous alloys and multi-principal-element composites has demonstrated significant potential in addressing complex challenges related to phase prediction, property optimization, and compositional design. By leveraging advanced ML algorithms such as artificial neural networks (ANNs), support vector machines (SVMs), random forests, and gradient boosting methods, researchers have achieved remarkable accuracy in predicting GFA, phase selection, and mechanical properties. These advancements not only enhance our understanding of the fundamental relationships between composition, structure, and properties but also accelerate the discovery and development of novel high-performance materials.

Looking ahead, several promising directions can further advance this field. First, the development of more sophisticated ML models, including deep learning architectures and hybrid approaches, could improve prediction accuracy and generalization across diverse alloy systems. Second, the integration of ML with high-throughput experimentation and computational methods, such as DFT and molecular dynamics (MD), can provide a more comprehensive understanding of material behavior. Third, addressing data quality and quantity issues through standardized data collection and sharing platforms will be critical for training robust and reliable models. Finally, the application of ML to real-time process optimization and alloy design in industrial settings holds great promise for translating laboratory discoveries into practical technologies.

In conclusion, the synergy between ML and materials science is poised to revolutionize the development of amorphous alloys and multi-principal-element composites, paving the way for innovative solutions to meet the growing demands of advanced engineering applications.

## Figures and Tables

**Table 1:** Machine learning model input features, data volume, and performance.

Researcher	Data Volume	Machine Learning Models	Features	Prediction Accuracy
Liu et al. [18]	3227	ANN (MLP)	13 features	ACC for multicomponent alloys (*n* > 3) prediction: 83%.
Islam et al. [19]	118	Neural network (NN)	(*VEC*), *S_id_*, *δ*, Δ*H_m_*, and Δ*χ*	Achieved a test set accuracy of 83% (particularly *S_id_*, *δ*, Δ*H_m_*, and Δ*χ*; reduced prediction accuracy by over 7%).
Zhou et al. [20]	601	ANN, SVM, KNN	11 features	All three ML algorithms achieved high prediction accuracy.
Huang et al. [21]	401	KNN, ANN, SVM	(*VEC*), *S_id_*, *δ*, Δ*H_m_*, and Δ*χ*	The KNN algorithm achieved an accuracy of 68.6%, the SVM algorithm achieved 64.3%, and the ANN algorithm achieved 74.3%.
Zhang et al. [22]	407	SVM	4 features	Achieved accuracy of 88.77–94.63% for amorphous phases, 95.67–97.87% for solid solution phases, and 96.03–98.63% for mixed solid solution and intermetallic phases.
Wu et al. [23]	321	MLP, ANN, GBDT	9 features	Achieved a test set accuracy of 98.3%

**Table 2:** Machine learning in model selection and data volume and performance.

Researcher	Data Volume	Machine Learning Models	Forecast Results
Xiong et al. [66]	442	SVM	*R*^2^ = 0.57
Deng et al. [67]	442	Random forest	*R*^2^ = 0.64
Xiong et al. [44]	6471	Symbolic regression and random forest model	The random forest model achieved the highest R value of 0.85
Ward et al. [49]	607	Random forest, decision tree, and ensemble models	The random forest model achieved 89%
Liu et al. [68]	6816	Random forest	The random forest model achieved 89%
Peng et al. [69]	810	Random forest	*R*^2^ = 0.682
Tripathi et al. [74]	410	Genetic algorithm	95.26%
Mastropietro et al. [75]	480	Multiple Linear Regression and Tree Boosting Model	*R* = 0.84 (after integrating two models)
Long et al. [79]	698	Six algorithms	The determination coefficient *R*^2^ of the decision tree model can reach 0.763

**Table 3:** Advantages and limitations of several common machine learning algorithms.

Algorithms	Advantages	Limitations	Applicable Scenarios
Traditional linear models (linear regression, logistic regression)	High computational efficiency, suitable for small sample data (material science data are usually limited). Strong interpretability, can analyze feature importance through coefficients. Suitable for exploring simple linear relationships (such as the preliminary association between alloy composition and GFA).	Unable to capture the complex nonlinear relationships of amorphous alloys (such as the synergistic effect of multi-component alloys). Sensitive to noise, assumes feature independence, and may ignore the interaction of physical and chemical features.	Preliminary screening of features or baseline models.
SVM	Stable performance in high-dimensional space (suitable for the feature space of multi-component alloys). Kernel techniques (such as RBF kernel) can handle nonlinear problems.	Complex parameter tuning (such as kernel function selection, regularization parameters). Lack of intuitive physical interpretability; difficult to guide material design. Low computational efficiency for large-scale data.	Small and medium-sized datasets, when it is necessary to balance accuracy and complexity.
Decision Tree and Random Forest	Naturally handle nonlinear relationships and are suitable for feature interactions of complex alloy systems. Provide feature importance ranking (such as key parameters such as atomic size difference and mixing entropy). Not sensitive to missing values and strong resistance to overfitting (random forest).	Single decision tree is prone to overfitting and needs to rely on ensemble methods (such as RF). Limited generalization ability for small sample data (needs to be combined with cross-validation).	Feature importance analysis and medium-sized dataset prediction (such as GFA prediction).
Gradient Boosting Machine (XGBoost, LightGBM)	High prediction accuracy, excellent performance in material science competitions. Automatically handle missing values and support parallel computing. Adjustable regularization term to avoid overfitting.	Complex hyperparameter tuning (learning rate, tree depth, etc.). High model complexity, weaker interpretability than random forest.	Scenarios with high precision requirements (such as mechanical property prediction).
Neural Networks (NN) and Deep Learning	Powerful nonlinear modeling capabilities, suitable for high-dimensional features (such as multi-component composition, process parameters). Deep learning models (such as CNN, graph neural network (GNN)) can process structural data (such as atomic neighborhood topology).	Requires a large amount of data (easy to overfit when material data is scarce). Black box model, poor interpretability, difficult to guide experimental design. Large computing resource consumption (such as GPU training).	Large-scale datasets or combined with transfer learning (such as pre-trained models).
Bayesian Optimization and Gaussian Process (GP)	Provide uncertainty estimates for prediction results (instructive for experimental design). Suitable for small sample optimization (such as iterative search of alloy composition).	High computational complexity; difficult to extend to high-dimensional features. Kernel function selection affects performance.	Experimental parameter optimization or active learning framework.
